# Dietary therapy of the herbal porridge improves the symptoms of functional dyspepsia: A randomized, double‐blind, placebo‐controlled, clinical trial

**DOI:** 10.1002/fsn3.3911

**Published:** 2024-01-07

**Authors:** Xin Wang, Meng‐Ao Liu, Jia He, Wen‐Zhi Zhao, Yi‐Xuan Wang, Liu‐Qing Yang, Tian‐Yuan Wang, Han‐Ping Shi, Ben‐Qiang Rao

**Affiliations:** ^1^ Departments of General Surgery, Beijing Shijitan Hospital Capital Medical University Beijing China; ^2^ Center of Nutrition and Metabolism of Cancer, Beijing Shijitan Hospital Capital Medical University Beijing China; ^3^ Key Laboratory of Cancer FSMP for State Market Regulation Beijing China; ^4^ Beijing International Science and Technology Cooperation Base for Cancer Metabolism and Nutrition Beijing China; ^5^ Departments of Critical Care Medicine, Beijing Shijitan Hospital Capital Medical University Beijing China; ^6^ Departments of Clinical Nutrition, Beijing Shijitan Hospital Capital Medical University Beijing China; ^7^ Departments of Emergency, Beijing Shijitan Hospital Capital Medical University Beijing China

**Keywords:** dietary therapy, epigastric symptoms, functional dyspepsia, herbal porridge, randomized controlled trial, traditional Chinese medicine

## Abstract

This study (ISRCTN17174559) aimed to explore the efficacy and safety of a kind of herbal porridge (Hou Gu Mi Xi) on the clinical symptoms of functional dyspepsia (FD). This was a single‐center, single‐dose, prospective, double‐blind, randomized controlled trial involving 64 participants with FD (35 cases and 29 controls) for 2 months of intervention and 1 month of follow‐up. The 7‐point Global Overall Symptom Scale (GOSS), 36‐Item Short Form Survey (SF‐36), and other indicators were assessed at baseline (day 0), at days 15, 30, and 60 of treatment, and at follow‐up 1 month after the end of the intervention. Many participants with FD achieved remission of their epigastric symptoms at follow‐up on the 90th day after treatment with herbal porridge compared to the placebo group (45.71% vs. 20.69%, *p* = .036). Furthermore, herbal porridge appeared to be effective in improving the quality of life of participants with FD, which was reflected in the rising SF‐36 scores for physical role, bodily pain, emotional role, and mental health. Although adverse events were reported, there was no overall difference in the number of adverse events between the two groups (*p* = .578). Herbal porridge is another effective and safe method for improving the symptoms and quality of life in patients with FD.

## Graphical abstract

This study (ISRCTN17174559) aimed to explore the efficacy and safety of a kind of herbal porridge on clinical symptoms of functional dyspepsia (FD), which was a single‐center, single‐dose, prospective, double‐blind, randomized controlled trial involving 64 participants of FD (35 cases and 29 controls) for 2 months of intervention and 1 month of follow‐up. In conclusion, herbal porridge provides another effective and safe method for improving the symptoms and quality of life in patients with FD.

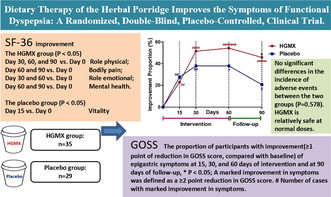

## 1 INTRODUCTION

According to statistics (El‐Serag & Talley, 2004), 30%–40% of adults often have symptoms centered on the upper abdomen, such as epigastric pain, discomfort, burning, early satiety (inability to finish a normal‐sized meal), postprandial fullness, belching, acid reflux, nausea, vomiting, and loss of appetite. However, after a thorough examination such as gastroscopy, it was found that only a few people had organic diseases of the stomach, such as gastroesophageal reflux, drug irritation, chronic disease, ulcer disease, and malignant tumors. Most of the remaining people were diagnosed with functional dyspepsia (FD) (Ford et al., 2010). An investigation showed that the overall prevalence rate of FD is 16% in the world (Ford et al., 2020); the prevalence is relatively high (10%–40%) in Western countries and 5%–30% in Asia (Enck et al., 2017). FD is more common in women (24.4%) than in men (16.6%), and its incidence increases significantly with age (Piessevaux et al., 2009). According to the diagnostic criteria for FD, symptoms need to be active in the past 3 months, with onset at least 6 months before diagnosis (Ford et al., 2020). The incidence of FD has increased in recent years. It is estimated that approximately 50% of people seek medical treatment for FD at some stage in their lives (Koloski et al., 2001).

FD can be divided into two subtypes: postprandial distress syndrome (PDS) and epigastric pain syndrome (EPS) (Enck et al., 2017). The former is characterized by early satiety and postprandial fullness, whereas the latter is characterized by upper abdominal pain and burning sensation. However, the pathogenesis of FD is not fully understood. It is currently associated with visceral hypersensitivity (Carbone & Tack, 2014), infection by *Helicobacter pylori* (Liu et al., 2021), acid exposure (Liu et al., 2021), slow gastric emptying (Carbone & Tack, 2014), smoking alcohol intake (Hongo, 2011), and mucosa‐associated microbiota (Shanahan et al., 2023). Surprisingly, psychological distress, particularly anxiety, and sleep disorders might also interact with the symptoms of FD (Aro et al., 2015). At present, the clinical treatment of FD is limited and mainly includes protecting the gastric mucosa, inhibiting acid, promoting gastrointestinal motility, eradicating *H. pylori*, taking antidepressants, and adopting other symptomatic treatments (Sayuk & Gyawali, 2020; Talley, 2017).

The diagnosis and treatment processes of FD are expensive, consume limited medical resources, and are often accompanied by therapeutic side effects (Camilleri & Stanghellini, 2013), which are not sustainable. The existence of long‐term symptoms has a negative impact on the quality of life of patients, but there is no organic lesion to target. Therefore, patients often seek benefits from food supplements or functional foods rather than the use of medications (Rosch et al., 2006). Dietary therapy of traditional Chinese medicine (TCM) combines the development of modern Chinese medicine with food for special medical purposes. However, it is different from medicines and ordinary diets and is a useful and safe supplement for the treatment of diseases. Under the guidance of TCM theory, which explores the properties, compatibility, preparation, eating methods, and function of medicinal foods, dietary therapy uses food to prevent and treat diseases and to promote rehabilitation and health. With the current high incidence of chronic diseases and popularization of the concepts of disease prevention and health preservation, research on dietary TCM therapy deserves extensive attention (Zou, 2016).

According to Chinese traditional medicine, FD belongs to the category of “Wei‐Pi” (Sin et al., 2021). Patients with “Wei‐Pi” often experience diminution or loss of appetite, epigastric pain, epigastric distention, ructus, belching, or feelings of fullness caused by “Qi” stagnation and disorders, “spleen–stomach” weakness, “spleen–stomach” deficiency, cold, and dampness–heat. Therefore, during “Wei‐Pi” treatment, “spleen” function should be effectively adjusted first, and the treatment should be based on invigorating “Qi” and “spleen”, and dispelling “dampness” (Sin et al., 2021). Shen Ling Bai Zhu San, a classic TCM formula (Tai Ping Hui Min He Ji Ju Fang, Song Dynasty [1102 AD]), can effectively improve plant nerve dysfunction and endocrine disorders caused by “spleen” deficiency and “Qi” stagnation to promote the secretion of digestive enzymes and increase the digestion speed of the stomach (Chen et al., 2018). Therefore, Shen Ling Bai Zhu San is used to treat FD with syndromes of spleen deficiency and “Qi” stagnation and is both safe and better than traditional Western medicine for relieving symptoms (Jia et al., 2018). Herbal porridge, a dietary TCM formula, is a modified form of Shen Ling Bai Zhu San (Chen et al., 2019). Previous research has revealed that similar to Shen Ling Bai Zhu San, herbal porridge is also effective and safe for patients with “spleen” deficiency syndrome after radical gastric cancer surgery (Zhou et al., 2019). Currently, there is a lack of evidence supporting the use of herbal porridge as a sustainable or auxiliary method to improve FD. Therefore, this study aimed to explore the efficacy and safety of herbal porridge on the clinical symptoms of FD and put forward some suggestions for dietary therapy with herbal porridge.

## 2 MATERIALS AND METHODS

### 2.1 Study design and ethics authorization

This study was a single‐center, single‐dose, prospective, double‐blind, randomized controlled trial (RCT) performed at Beijing Shijitan Hospital, Capital Medical University (Beijing, China), a comprehensive hospital that combines medical, teaching, and research domains. This study aimed to investigate the efficacy and safety of herbal porridge in the treatment of FD. The protocol was registered in the International Standard Randomized Controlled Trial Number (ISRCTN) Registry (Number: ISRCTN17174559) and was performed according to the Standard Protocol Items: Recommendations for Interventional Trials (SPIRIT) guidelines (Chan et al., 2013). This research scheme followed the Declaration of Helsinki and was approved by the Clinical Research Ethics Committee of Beijing Shijitan Hospital.

### 2.2 Participant recruitment and eligibility criteria

First, patients with FD were recruited from the community and Beijing Shijitan Hospital through health education courses or advertisements, in which the content and purpose of the intervention were fully explained. Inclusion criteria included: ① age 18–80 years, no sex restrictions; ② patients who met the Rome III diagnostic criteria for FD (the absence of organic dyspepsia was confirmed by endoscopy); ③ patients whose gastroscopy showed normal results within the past year, with no other organic lesions found in the gastrointestinal tract; ④ no mental illness (Mini Mental State Questionnaire, MMSE ≥20); ⑤ ability to take food orally; ⑥ voluntarily agreed to accept the intervention and signed the informed consent; and ⑦ compliance with treatment completion and follow‐up. Exclusion criteria included: ① patients with severe cognitive impairment, abnormal electrocardiogram, organic digestive tract disease, a history of gastrointestinal surgery, diabetes, malignant tumors, and other diseases; ② taking traditional Chinese medicine preparations; ③ pregnant or breast‐feeding women, or planning to become pregnant within 6 months; ④ a history of allergic reactions related to Chinese medicine or rice paste; ⑤ impairment of liver and kidney function (total bilirubin, alanine aminotransferase, or aspartate aminotransferase >2 times the upper limit of normal; and serum creatinine >2 times the upper limit of normal); ⑥ taking drugs that affect digestive function (such as gastrointestinal motility drugs, anti‐*Helicobacter pylori* drugs, antidepressants, antianxiety drugs, or acid‐suppressing drugs, such as proton pump inhibitors (PPIs) and histamine type 2 (H2) receptor blockers, etc.) that were taken within the past month or during the study period; ⑦ place of residence is too far away or the participant is unwilling or unable to complete the return visit; and ⑧ a positive *H. pylori* test by Carbon‐13 urea breath test at the time of screening.

### 2.3 Sample size

The main objective of this trial was to assess the effects of herbal porridge compared to placebo on the proportion of participants with improvement in overall symptoms. Therefore, sample size estimation was based on the formula for a superior design (Zhong, 2009). Given that there are no previous studies available for reference, we estimated a *p* of .2 (total effective rate receiving placebo) and a P0 of 0.5 (total effective rate receiving the herbal porridge), according to the results of our preliminary experiment and expert opinions.

In addition, we adopted a type I error (a) of 0.05, a type II error (b) of 0.2, and a dropout rate of 15%. A sample size of at least 74 participants was included.

### 2.4 Randomization and intervention

Study recruitment was performed from May 1, 2020, to December 31, 2021. A total of 122 patients with symptomatic FD were included in the detailed process of the trial and were asked whether they wanted to participate. According to the inclusion and exclusion criteria, finally, a total of 80 prospective participants were included in the study. Using a computer‐generated random number table, odd numbers were allocated to the intervention group and even numbers were allocated to the control group. All eligible participants were randomly assigned to two groups to receive either herbal porridge or a placebo (ordinary porridge) in a 1:1 ratio. Data analysts and participants were blinded to the group assignments in this trial.

Herbal porridge and a matching placebo were manufactured and provided by Jiangzhong Food Therapy Technology Co., Ltd. The herbal porridge is a kind of multigrain breakfast cereal with natural TCM ingredients, called “Hou Gu Mi Xi,” modified from Shen Ling Bai Zhu San. The herbal porridge contains Ren Shen (ginseng), Fu Ling (Poria), Shan Yao (yam), Lian Zi (lotus seed), Bai Bian Dou (white hyacinth bean), Yi Yi Ren (coix seed), Sha Ren (amomum fruit), Ju Pi (orange peel), Jie Geng (*Platycodon grandiflorum*), Gan Cao (*Radix glycyrrhizae*), Jing Mi (Japonica rice), and Yan Mai (oats). The placebo, which only provided energy and nutrition, consisted of starch, purple potato juice, and oats and was identical to the herbal porridge in terms of packaging, shape, and size, but had slight differences in taste and smell, which were difficult to differentiate. The participants had the right to cease participation at any time. Sufficient time was given to the participants to read the consent form, ask questions, and make the decision to sign. Subsequently, the researchers collected baseline information, including demographic characteristics, the 7‐point Global Overall Symptom Scale (GOSS), smoking history, alcohol consumption, general health condition, dietary intake, sleep quality, exercise habits, the 36‐Item Short Form Survey (SF‐36), body mass index (BMI), bodyweight, and other physical measurement indicators. Before starting the intervention, the researchers provided health education to the participants and face‐to‐face guidance on the usage, dosage, and precautions regarding the porridge. Porridge was distributed at the beginning of the first month. Without changing their normal diet, participants were instructed to consume either one packet (30 g) per day of herbal porridge or one packet (30 g) per day of placebo, taken orally for a 2‐month intervention period under the supervision of the researchers via WeChat.

### 2.5 Efficacy and safety outcome measures

The participants attended investigations, including GOSS, SF‐36, BMI, and other physical measurement indicators, at 15th, 30th, and 60th days after treatment, and follow‐up at 1 month after the expiration of the intervention, including GOSS, SF‐36, BMI, other physical measurement indicators, feedback, and feelings about the entire trial. The questionnaire consisted of a WeChat answer sheet. Participants were not required to visit the hospital to complete each visit at any time point, except for the baseline investigation. Participants were requested to take and submit pictures of any remaining porridge packages at the end of the 2‐month trial as an indicator of study compliance and completion. Participants who dropped out or withdrew from the trial, and their reasons for doing so, were recorded on a case report form. Any adverse events (AEs) and severe AEs (SAEs) were monitored throughout the trial period using a standard adverse event case report form.

The 7‐Point Global Overall Symptom Scale (GOSS) (Veldhuyzen van Zanten et al., 2006) is a well‐validated measure for assessing the intensity of overall gastric symptoms in patients with FD over a specified period. The score ranges from 1 to 7 points and higher scores indicate more severe symptoms, as follows: 1. no discomfort or problems; 2. slight problems (which can be easily ignored without effort); and 3. mild problems (can be ignored with effort); and 4. moderate problems (cannot be ignored but do not influence daily activities); 5. moderately severe problems (cannot be ignored and occasionally limit daily activities); 6. severe problems (cannot be ignored and often limits concentration on daily activities); and 7. very severe problems (cannot be ignored, markedly limit daily activities, and often require rest). The 36‐item Short Form Health Survey questionnaire (SF‐36) (Lins & Carvalho, 2016) is a universal instrument used to evaluate health‐related quality of life. In addition to the overall health change index, the SF‐36 measures eight domains: physical functioning (PF), role physical (RP), bodily pain (BP), and general health (GH), which constitute the physical dimension SF‐36; and vitality (VT), social functioning (SF), role emotional (RE), and mental health (MH), which constitute the mental dimension of the SF‐36. Higher scores indicate better health outcomes in patients with FD.

### 2.6 Statistical analysis

Categorical variables were presented as frequency or number, whereas continuous variables were presented as mean ± standard deviation. The chi‐square test or Fisher's exact test was used to compare categorical variables among study groups, as appropriate, while comparisons of continuous data between the two groups were performed using the *t*‐test. A binomial logistic regression analysis was conducted to examine the potential determinants of treatment efficacy. A two‐sided *p* value of <.050 was considered statistically significant. All data were analyzed using the SPSS software (version 19.0; IBM Corp., Armonk, NY, USA) and displayed using GraphPad Prism 7 (GraphPad Software Inc., La Jolla, CA, USA).

## 3 RESULTS

### 3.1 Participant recruitment and baseline characteristics

**FIGURE 1:**
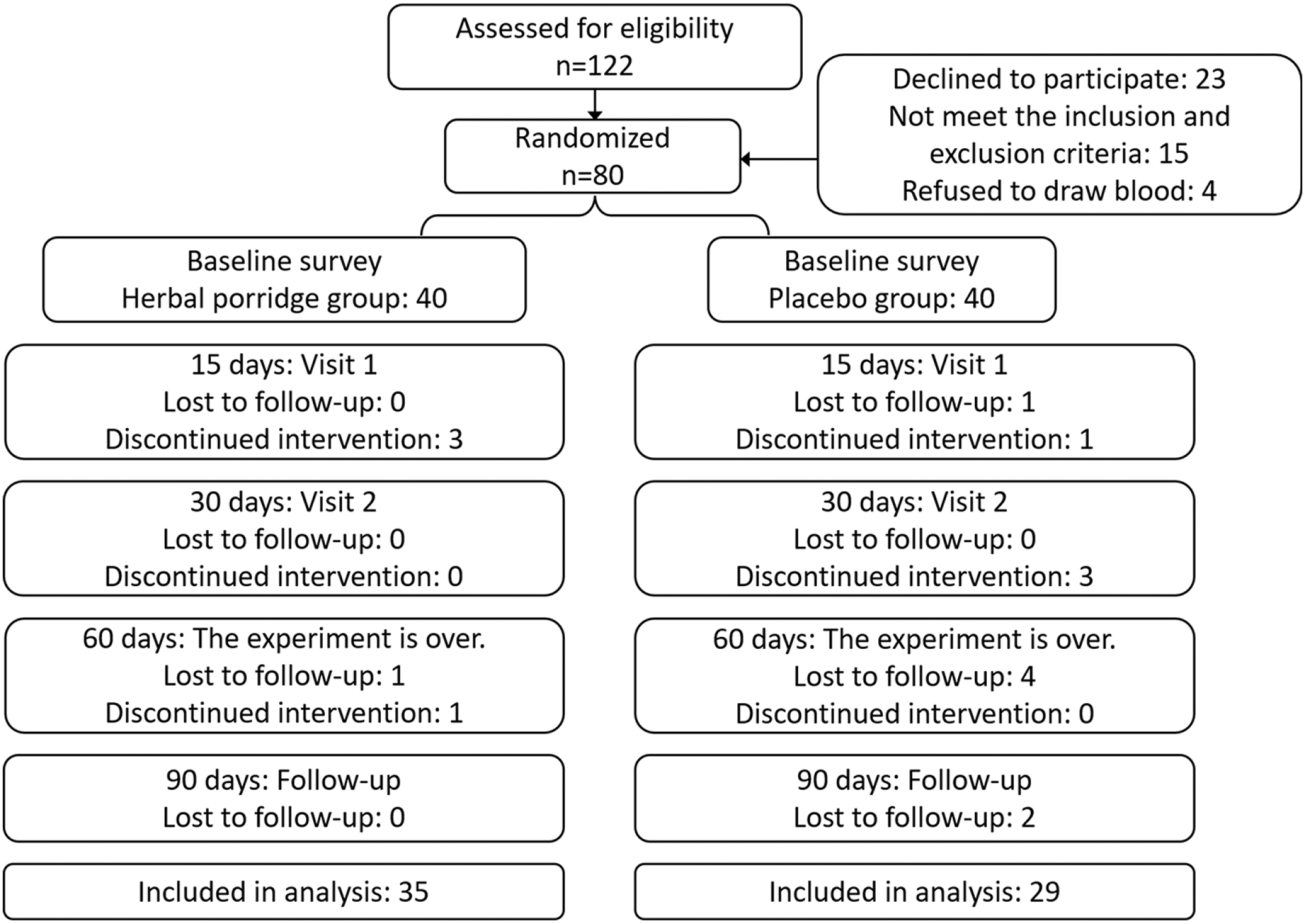
Flow diagram illustrating the number of participants in each group throughout the study.

Of the 122 participants with FD who were screened from the community or who were outpatients, 23 declined to participate, 15 did not meet the inclusion and exclusion criteria (*H. pylori* positive, *n* = 9; significantly abnormal gastrin, *n* = 1; severe diabetes, *n* = 2; liver and kidney dysfunction, *n* = 1; antidepressant treatment, *n* = 1; and pregnancy, *n* = 1), and 4 refused to draw blood. Eighty participants underwent randomization, which was consistent with the sample size calculation, and 80.0% of them completed the study. During the trial, eight (one in the herbal porridge group and seven in the placebo group) were lost to follow‐up (data not shown); four (three in the herbal porridge group and one in the placebo group) dropped out because of an unbearable taste; one participant was too busy to take the intervention and dropped out from the herbal porridge group; and two participants dropped out from the placebo group due to taking drugs and another dropped out from the placebo group due to studying abroad. Therefore, 64 patients with symptomatic FD (35 in the herbal porridge group and 29 in the placebo group) completed the clinical trial, and the results were analyzed. A flowchart of the participant screening process is presented in Figure 1.

The mean age of the participants was 35.44 ± 10.77 years, and the mean BMI (kg/m^2^) was 21.53 ± 2.46. There were 23 men (35.94) and 41 women (64.06%) in this study. The baseline characteristics of the participants with FD are presented in Table 1. No significant differences were found between the herbal porridge and placebo groups in terms of baseline characteristics, including sex, age, smoking status, alcohol consumption, exercise habits, and sleep quality (*p* > .05), except for BMI (*p* = .013).

**TABLE 1:** Demographic and clinical characteristics of participants with FD.

Characteristics	Herbal porridge group (*n* = 35)	Placebo group (*n* = 29)	All (*n* = 64)	*p* Value
Sex (male)^a^	11 (31.43)	12 (41.38)	23 (35.94)	.409
Age in years^b^	35.57 (11.86)	35.28 (9.49)	35.44 (10.77)	.914
BMI (kg/m^2^)^b^	20.84 (2.25)	22.37 (2.47)	21.53 (2.46)	.013*
Smoking status (Yes)^a^ ^,^ ^c^	5 (14.29)	8 (27.59)	13 (20.31)	.188
Alcohol consumption (Yes)^a^ ^,^ ^d^	6 (17.14)	9 (31.03)	15 (23.44)	.192
Exercise habits (Yes)^a^ ^,^ ^e^	27 (77.14)	20 (68.97)	47 (73.44)	.461
Sleep quality (Good)^a^ ^,^ ^f^	12 (34.29)	9 (31.03)	21 (32.81)	.783
Going to bed late (Yes)^a^ ^,^ ^g^	8 (22.86)	7 (24.14)	15 (23.44)	.904
Having breakfast (Often)^a^ ^,^ ^h^	32 (91.43)	25 (86.21)	57 (89.06)	.792
Taking meals on time (No)^a^ ^,^ ^i^	6 (17.14)	7 (24.14)	13 (20.31)	.489
Advocation in dietary therapy (Yes)^a^ ^,^ ^j^	20 (57.14)	15 (51.72)	35 (54.69)	.665
Type of FD (1)^a^ ^,^ ^k^	24 (68.57)	21 (72.41)	45 (70.31)	.738
Any treatment for FD (Yes)^a^	13 (37.14)	8 (27.59)	21 (32.81)	.418
Eaten TCM for FD before (Yes)^a^	4 (11.43)	3 (10.34)	7 (10.94)	1.000

Abbreviations: BMI, body mass index; FD, functional dyspepsia.

^a^ Categorical variables are presented as number (percentage).

^b^ Continuous variables are presented as mean (standard deviation).

^c^ The standard is smoking in the past week.

^d^ The standard is drinking in the past week.

^e^ The standard is more than 1 h of exercise in the past week.

^f^ The standard is good sleep quality >50% in the past 3 months.

^g^ The standard is going to bed after midnight >50% in the past 3 months.

^h^ The standard is eating breakfast >50% in the past 3 months.

^i^ The standard is irregular meals >50% in the past 3 months.

^j^ The standard is belief in dietary therapy all the times.

^k^ 1. Postprandial distress syndrome, PDS; 2. Epigastric pain syndrome, EPS.

* *p* < .05.

### 3.2 Symptoms of FD at baseline

**FIGURE 2:**
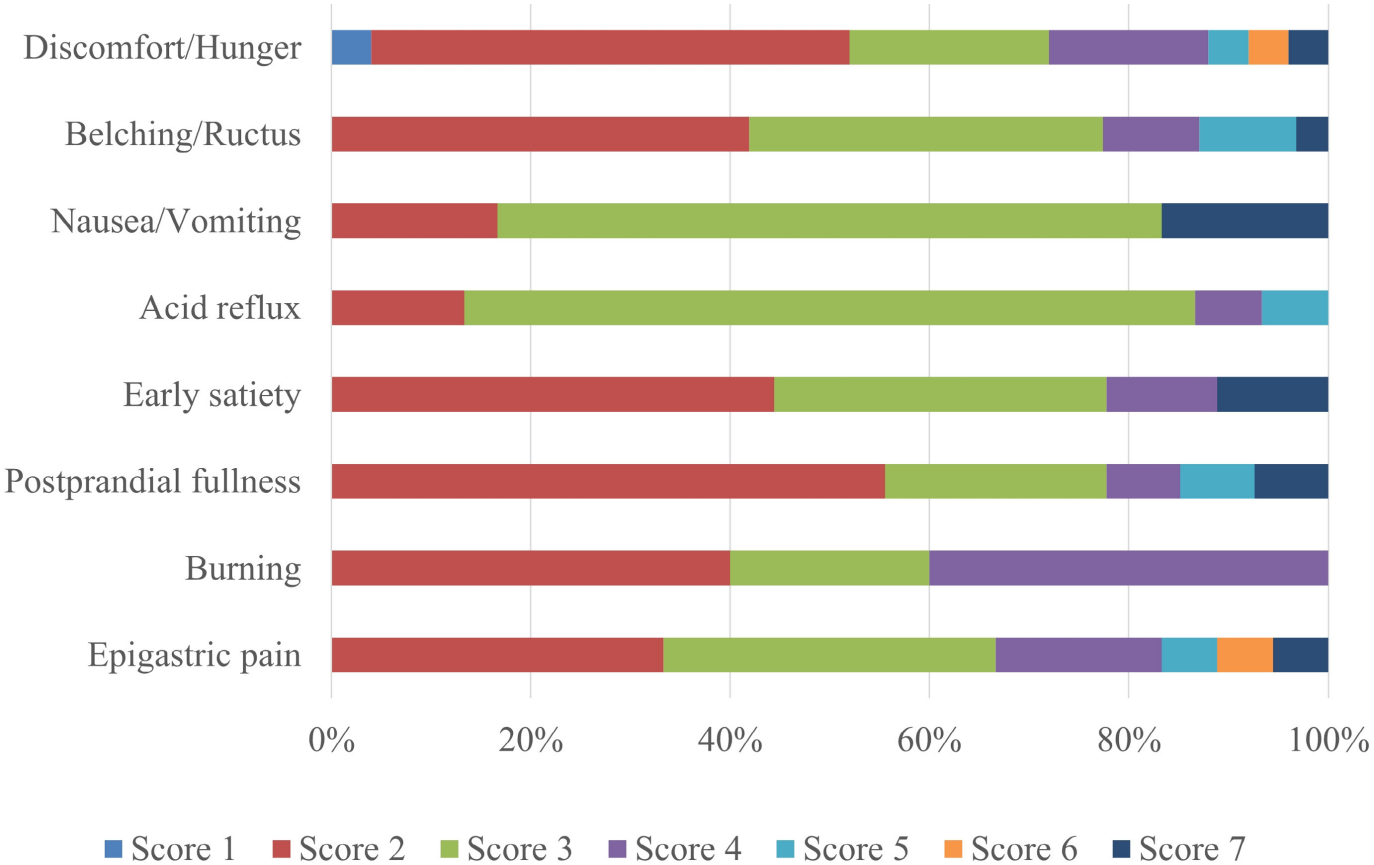
Upper gastrointestinal symptoms in 64 participants with FD at baseline scored on the 7‐Point Global Overall Symptom Scale (GOSS).

In our study, most participants with FD had a GOSS score of 2–3 points. A score of 2 accounted for 42.19% and a score of 3 accounted for 32.81% of all participants. The mean GOSS score for upper gastrointestinal symptoms was 3.0 at baseline. In terms of all symptoms, belching/ructus was the most common (reported by 48.44% of the participants), followed by postprandial fullness (42.19%) and discomfort/hunger (39.06%). Acid reflux was the most frequent symptom in GOSS ≤3; and burning was the most frequent symptom in GOSS >3, as shown in Figure 2. Overall, 73.44% of patients had more than one type of predominant symptom.

### 3.3 The improvement rate of symptoms in FD participants between the herbal porridge group and the placebo group during the intervention

**FIGURE 3:**
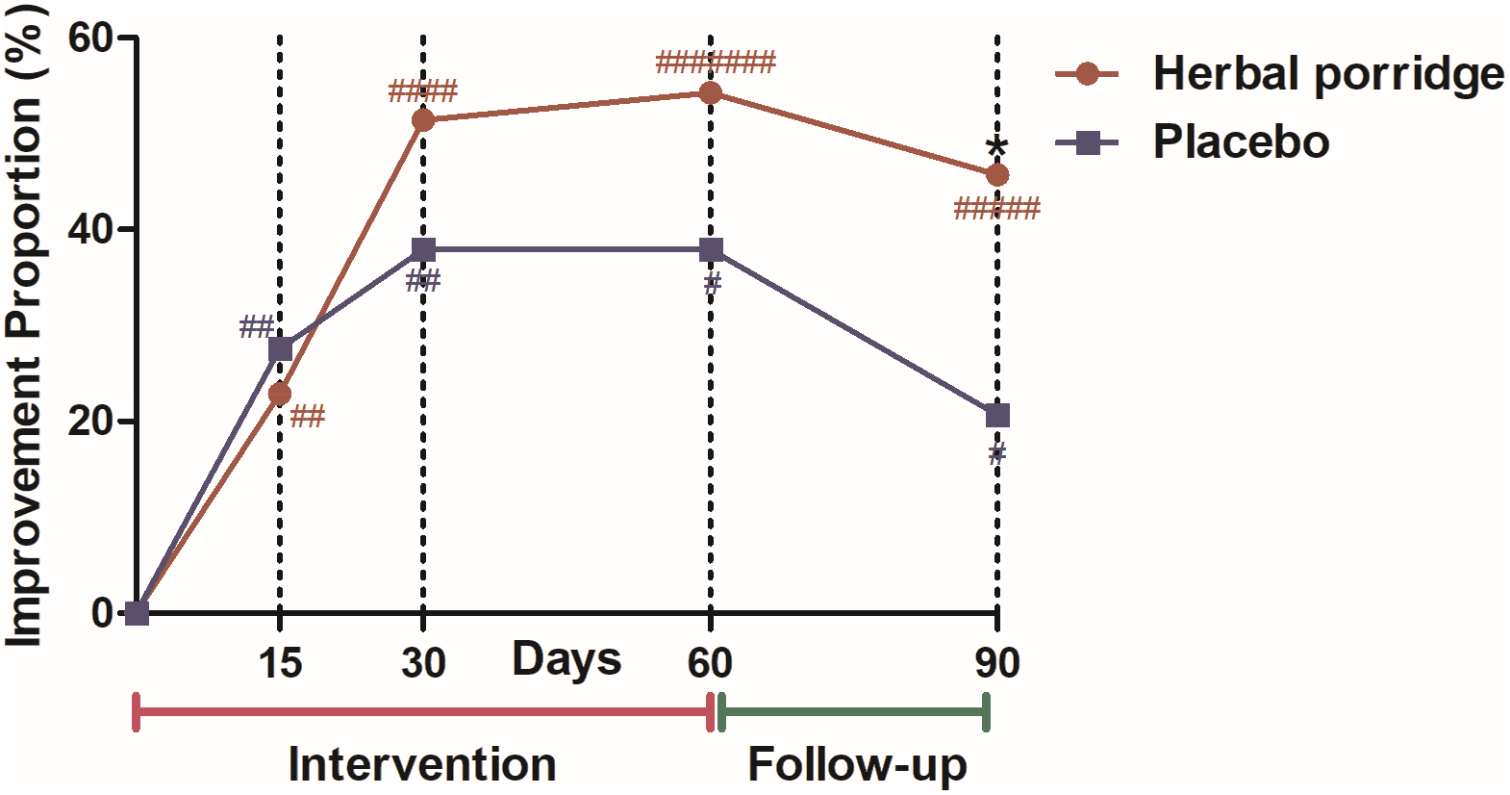
The proportion of participants with improvement of epigastric symptoms at 15, 30, and 60 days of intervention and 90 days of follow‐up. Symptoms were scored using the GOSS scale, and the improvement of symptoms was defined as a ≥1 point reduction in GOSS score, compared with baseline (day 0), *p < .05, herbal porridge versus placebo; a marked improvement in symptoms was defined as a ≥2 point reduction in GOSS score, compared with baseline (day 0). #Number of cases with marked improvement in symptoms.

Bulleted lists look like this: On the 15th, 30th, and 60th days of treatment, compared with baseline (day 0), the proportion of participants with improvement in overall symptoms (a GOSS score decrease ≥1 point) in the herbal porridge group was 22.86%, 51.43%, and 54.29%, respectively, and the proportion of participants with improvement in overall symptoms in the placebo group was 27.59%, 37.93%, and 37.93%, respectively. The proportion of participants with an improvement in overall symptoms after 30 and 60 days of treatment was higher in the herbal porridge group than in the placebo group; however, the difference was not statistically significant (*p* > .05). At follow‐up on the 90th day (1 month after treatment ended), the proportion of participants with improvement in overall symptoms in the herbal porridge group was significantly greater than that in the placebo group (45.71% vs. 20.69%, *p* = .036) (Figure 3).

A ≥2 points reduction in GOSS score, compared with baseline (day 0), was defined as a marked improvement in epigastric symptoms. The number of participants with marked improvement was seven in the herbal porridge group and four in the placebo group after 60 days of intervention. Twenty percent of the participants showed marked improvement in GOSS scores after receiving treatment (Figure 3).

### 3.4 The effect of the intervention on symptom improvement by subtype of FD

Binomial logistic regression analysis was performed using the presence or absence of improvement in FD at 60th and 90th days after intervention (as the dependent variable), and the independent variables included treatment group, sex, age, BMI, smoking status, alcohol consumption, exercise habits, sleep quality, going to bed late, having breakfast, eating meals on time, and type of FD (Table 2).

**TABLE 2:** Demographic characteristics and their association with symptom improvement of FD at day 60 after intervention and day 90 of follow‐up using binomial logistic regression analysis.

Variables^b^	60 days		90 days	
	Adjusted OR (95% CI)^a^	*p* Value	Adjusted OR (95% CI)	*p* Value
Group				
Placebo	1.00	.357	1.00	.042
Herbal porridge	1.777 (0.522–6.042)		3.327 (1.046–10.586)	
Sex				
Female	1.00	.451	1.00	.721
Male	0.540 (0.109–2.682)		0.725 (0.124–4.235)	
Age				
Continuous	1.004 (0.939–1.073)	.914	1.036 (0.966–1.111)	.322
BMI (kg/m^2^)				
Continuous	0.964 (0.693–1.341)	.826	0.769 (0.541–1.093)	.144
Smoking status				
No	1.00	.648	1.00	.221
Yes	1.541 (0.240–9.889)		3.643 (0.460–28.820)	
Alcohol consumption				
No	1.00	.557	1.00	.407
Yes	0.613 (0.120–3.140)		0.456 (0.071–2.926)	
Exercise habits				
No	1.00	.621	1.00	.640
Yes	1.466 (0.322–6.669)		0.689 (0.145–3.283)	
Sleep quality				
Good	1.00	.245	1.00	.482
Poor	2.100 (0.601–7.333)		1.635 (0.415–6.436)	
Going to bed late				
No	1.00	.448	1.00	.740
Yes	0.553 (0.120–2.556)		1.317 (0.259–6.696)	
Having breakfast				
No	1.00	.540	1.00	.288
Yes	2.284 (0.162–32.161)		4.356 (0.289–65.707)	
Taking meals on time				
No	1.00	.490	1.00	.192
Yes	0.530 (0.087–3.221)		0.281 (0.042–1.896)	
Type of FD				
2	1.00	.007	1.00	.053
1	0.197 (0.060–0.647)		0.316 (0.098–1.016)	

Abbreviations: BMI, body mass index; CI, confidence interval; FD, functional dyspepsia; OR, odds ratio.

^a^ Models were adjusted for group, sex, age (as a continuous variable), BMI (as a continuous variable), smoking status, alcohol consumption, exercise habits, sleep quality, going to bed late, having breakfast, taking meals on time, and type of FD.

^b^ The standard of various parameters was displayed in Table 1.

After combining all variables, binomial logistic regression analysis showed that greater symptom improvement occurred in participants treated with herbal porridge than in those who received the placebo on the 90th day of follow‐up (OR = 3.327, 95% CI: 1.046–10.586, *p* = .042). Moreover, participants with PDS were less likely than participants with EPS to show symptom improvement after 60 days (OR = 0.197, 95% CI: 0.060–0.647, *p* = .007) and 90 days of intervention (OR = 0.316, 95% CI: 0.098–1.016, *p* = .053) (Table 2).

### 3.5 The quality of life in FD participants indicated by the SF‐36 questionnaire between the herbal porridge group and the placebo group during the intervention

Before the intervention, there was no statistically significant difference between the herbal porridge and placebo groups in terms of SF‐36 scores, and the two groups were comparable. Paired‐sample *t*‐tests were used. Table 3 shows the changes in all outcomes of SF‐36 scores from baseline (day 0) to day 90 in both groups.

**TABLE 3:** The SF‐36 questionnaire between the herbal porridge group and the placebo group during the intervention (Mean ± Standard deviation).

Group (FD)	Time (days)	The overall health change	Physical dimension			
			Physical functioning	Role physical	Bodily pain	General health
Herbal porridge	0	53.57 ± 25.10	92.86 ± 9.80	77.86 ± 34.18	72.66 ± 18.69	65.63 ± 20.60
	15	54.29 ± 23.86	93.57 ± 11.02	84.29 ± 30.37	78.06 ± 15.76	61.71 ± 18.48
	30	54.29 ± 25.35	95.00 ± 7.17	89.29 ± 25.93*	78.69 ± 16.84	64.03 ± 18.26
	60	55.00 ± 24.85	95.43 ± 6.68	90.71 ± 25.06*	79.37 ± 18.89*	64.66 ± 18.56
	90	54.29 ± 25.35	95.29 ± 6.85	92.14 ± 20.80*	79.49 ± 18.73*	64.40 ± 18.29
Placebo	0	48.28 ± 29.83	90.34 ± 16.74	89.66 ± 25.46	73.31 ± 19.26	59.10 ± 21.33
	15	46.55 ± 31.85	93.10 ± 14.97	88.79 ± 25.52	72.76 ± 18.84	57.10 ± 19.57
	30	46.55 ± 31.85	92.76 ± 15.44	87.93 ± 25.55	72.62 ± 18.69	57.38 ± 19.70
	60	46.55 ± 31.85	93.45 ± 15.07	89.66 ± 23.64	72.48 ± 19.85	57.72 ± 19.47
	90	46.55 ± 31.85	92.59 ± 15.39	87.93 ± 25.55	72.79 ± 18.70	57.55 ± 19.52

			**Mental dimension**			
			**Vitality**	**Social functioning**	**Role emotional**	**Mental health**
Herbal porridge	0		68.71 ± 13.63	83.43 ± 18.62	77.14 ± 36.85	65.03 ± 16.67
	15		67.43 ± 16.91	85.71 ± 16.50	83.81 ± 27.26	66.74 ± 16.05
	30		69.14 ± 14.12	84.86 ± 16.52	89.52 ± 23.94*	68.57 ± 13.27
	60		69.43 ± 14.64	88.00 ± 13.46	91.43 ± 21.91*	69.37 ± 13.05*
	90		69.71 ± 15.19	87.43 ± 14.21	90.48 ± 25.01	69.37 ± 12.87*
Placebo	0		61.21 ± 18.40	87.24 ± 15.56	83.91 ± 24.59	64.69 ± 17.44
	15		63.79 ± 19.67*	84.83 ± 17.45	85.06 ± 22.86	65.24 ± 19.51
	30		63.45 ± 20.00	85.17 ± 17.65	85.06 ± 22.86	65.38 ± 19.69
	60		63.28 ± 21.31	85.17 ± 17.65	82.76 ± 26.16	65.79 ± 19.70
	90		62.24 ± 17.66	85.86 ± 16.15	81.61 ± 26.10	64.83 ± 20.24

Abbreviation: FD, functional dyspepsia.

* Compared with baseline (day 0), *p* < .05.

At 30, 60, and 90 days, the herbal porridge group showed a significantly greater improvement in role physical compared to baseline (*p* = .047, .040, and .018, respectively); At 60 and 90 days, the herbal porridge group showed a significantly greater improvement in bodily pain than at baseline (*p* = .03 and .040, respectively). At 30 and 60 days, the herbal porridge group showed a significantly greater improvement in role emotional than at baseline (*p* = .040; .030, respectively). At 60 and 90 days, the herbal porridge group showed a significantly greater improvement in mental health than at baseline (*p* = .013 and .012, respectively). From baseline to follow‐up, the scores of the herbal porridge group did not change significantly in overall health change, physical functioning, general health, vitality, and social functioning (*p* > .05). The placebo group also showed greater improvement in the vitality score between baseline and day 15 (*p* = .037); however, no statistical significance was reached in any of the other dimensions during the intervention and follow‐up in the placebo group (*p* > .05). Table 3 presents the results of the study.

### 3.6 Safety assessment and satisfaction survey

A safety assessment was performed on 80 participants who had ingested porridge at least once. During the study, 13 adverse events were reported in the herbal porridge group (one participant developed a mild allergy on day 2 after treatment; one participant complained of an increase in constipation and abdominal pain during the intervention; two participants complained of slight diarrhea during the intervention; one participant complained of frequent dryness in the mouth while taking the porridge; one participant complained of transient abdominal distension while taking the porridge; one participant felt dry heat during the intervention; and one participant reported serious adverse events, including diarrhea, dizziness, insomnia, and palpitations, a few days after taking the porridge; after requesting a detailed history from the participant, we discovered that her symptoms had occurred in the past and were unrelated to the study). Moreover, five adverse events were reported in the placebo group (one participant developed a mild allergy on day 5 of treatment, one participant complained that his acid reflux worsened during the intervention, one participant reported transient palpitations and occasional constipation after taking the porridge, and two participants felt too full after taking the porridge, leading to loss of appetite), as shown in Table 4. Statistical analysis showed no significant difference in the incidence of adverse events between the two groups (*p* = .578). The adverse effects were transient, tolerable, and improved without treatment.

**TABLE 4:** Safety assessment and satisfaction survey of the two kinds of porridge.

Index	Herbal porridge group (*n* = 35)		Placebo group (*n* = 29)		*p* Value
	*n*	%	*n*	%	
AE	8	22.86	5	17.24	.578
SAE	1	2.86	0	0.00	1.000
Satisfaction	28	80.00	16	55.17	.033*

Abbreviations: AE, adverse events; SAE, serious adverse events.

* *p* < .05.

In terms of satisfaction rates, participants praised both the herbal and placebo porridges (Table 4). Overall satisfaction with the herbal porridge group was higher, reaching up to 80% satisfaction. Due to the high quality of ordinary porridge, participants in the control group also reported a high satisfaction rate (55.17%). The curative satisfaction of the herbal porridge group was significantly higher than that of the placebo group, indicating the efficacy of herbal porridge in improving the symptoms of FD.

## 4 DISCUSSION

To the best of our knowledge, this is the first registered randomized, double‐blind, placebo‐controlled clinical trial to clarify the efficacy and safety of herbal porridge compared with a placebo using the GOSS and SF‐36 scales. According to our observations, more participants with FD achieved remission of their epigastric symptoms at follow‐up on the 90th day after the 2‐month herbal porridge treatment period compared to the placebo group (Figure 3). The effect of the herbal porridge treatment was approximately 3.327 times higher than that of the placebo in terms of symptom improvement in FD at 90 days (Table 2). Although the difference between the two groups in terms of the proportion of participants with a GOSS decrease at day 60 of the intervention was not statistically significant, we were able to see a trend of symptomatic remission at 30 and 60 days after the herbal porridge treatment (Figure 3), which also provided data on the short‐term efficacy of herbal porridges. Binomial logistic regression analysis revealed that the FD type affected symptom improvement outcomes. Herbal porridge treatment was more effective in patients with EPS; however, symptoms of PDS may be more difficult to relieve with herbal porridge treatment (Table 2). Furthermore, FD significantly decreased the quality of life (Table 3). Herbal porridge, as a dietary formula based on TCM theory, seems to be effective in improving the quality of life of patients with FD, as diagnosed by the Rome III criteria, during the intervention and follow‐up, and this was reflected in the increased SF‐36 scores for role physical, bodily pain, role emotional, and mental health assessments (Table 3). Given that the placebo (ordinary porridge) consisted of starch, purple potato juice, and oats and was taken regularly according to the study protocol, consumption of the placebo may have had favorable effects on the FD participants in the placebo group with a history of poor dietary habits, which may have had positive effects on the vitality score of the SF‐36 and led to satisfaction of symptomatic relief in the results (Tables 3 and 4). Although adverse events were reported, there was no overall difference in the number of adverse events between the two groups (Table 4). These findings suggest that the herbal porridge is relatively safe at normal doses.

In clinical practice, there are no effective therapeutic approaches for FD because of the multifactorial etiology and recrudescent characteristics of the disease, and a lack of validated Western medicines. Traditional Chinese herbal therapies often lack a clear mechanism of action but have shown beneficial effects on the symptoms of FD (Enck et al., 2017; Ha, Jeong, et al., 2023; Ha, Ko, et al., 2023; Kim et al., 2023). For instance, after thousands of years of application and research, the use of Shen Ling Bai Zhu San as a classic ancient prescription of traditional Chinese medicine has been validated in terms of efficacy and safety for FD treatment (Zhang et al., 2019). However, Shen Ling Bai Zhu San has side effects and is difficult to popularize. Dietary TCM formula, as a special herbal therapy, is currently becoming a major concern in the area of TCM and sustainable diet, owing to the safety attributes of the diet and the therapeutic attributes of the herb. All herbs of the herbal porridge should be used together, giving full play to the effects of invigorating the spleen and stomach and regulating the flow of “Qi” to alleviate symptoms (Chen et al., 2018). After removing Bai Zhu (*Atractylodes macrocephala* Koidz), the herbal porridge was on the official list of foods designated by the Ministry of Health of China, which did not change the main effects of Shen Ling Bai Zhu San (Zhou et al., 2019). From TCM prescriptions to pure food and the sustainability of diets, the incidence of adverse reactions has obviously decreased, with herbal porridge treatment adding a certain degree of nutrition, safety, health, environmental protection, cultural confidence, and economy. Moreover, according to modern pharmacological research on Shen Ling Bai Zhu San, we speculated that herbal porridge may stimulate the production of gastroprotective digestive enzymes that participate in the alleviation of epigastric pain or correct vegetative system dysfunction to promote gastric emptying, thereby alleviating the symptoms of stomach discomfort after eating (Zhang et al., 2019).

However, this study had some limitations. The treatment course and follow‐up duration of this RCT were short, and the patient sample size was insufficient. Owing to these factors, it was not possible to determine the lasting and exact effects of the treatment regimen. Therefore, future multicenter studies with larger sample sizes and long‐term interventions are required to confirm the therapeutic effects of the herbal porridge. In the trial, participants needed to provide normal results from a gastroscope examination within the past 1 year; a baseline gastroscope examination was not included as part of the data collection owing to limited funding. Therefore, the study samples were at high risk of selection and performance bias because an exact diagnosis of FD at baseline was not available. The participants in this study were relatively young and mostly had very mild symptoms (the majority of participants with FD scored 2–3 points on the GOSS). There is a potential for selection bias in this trial because patients with severe symptoms or those who tended to reject diet therapy were switched to drug therapy.

## 5 CONCLUSIONS

This RCT is the first to assess the use of a dietary TCM formula, herbal porridge, for the management of FD. Herbal porridge is another effective and safe method for improving the symptoms and quality of life of patients with FD. Therefore, it could be considered a good candidate for FD treatment in clinical settings. The results of this RCT provide additional evidence for establishing guidelines and policies to facilitate the reasonable use and sustainability of dietary TCM formulas for the treatment of FD.

## AUTHOR CONTRIBUTIONS

**Xin Wang:** Conceptualization (equal); data curation (equal); formal analysis (equal); funding acquisition (equal); investigation (equal); methodology (equal); resources (equal); software (equal); supervision (equal); validation (equal); visualization (equal); writing – original draft (equal); writing – review and editing (equal). **Meng‐Ao Liu:** Conceptualization (equal); data curation (equal); investigation (equal); writing – original draft (equal). **Jia He:** Conceptualization (equal); formal analysis (equal). **Wen‐Zhi Zhao:** Conceptualization (equal); data curation (equal); formal analysis (equal); methodology (equal); software (equal); writing – review and editing (equal). **Yi‐Xuan Wang:** Conceptualization (equal); investigation (equal); supervision (equal); writing – review and editing (equal). **Liu‐Qing Yang:** Data curation (equal); investigation (equal). **Tian‐Yuan Wang:** Data curation (equal); resources (equal). **Han‐Ping Shi:** Project administration (equal); supervision (equal). **Ben‐Qiang Rao:** Conceptualization (equal); methodology (equal); project administration (equal); supervision (equal); writing – review and editing (equal).

## FUNDING INFORMATION

This research was funded by the Chinese Institute of Food Science and Technology, Food Science and Technology Fund (grant number 2019‐07).

## CONFLICT OF INTEREST STATEMENT

The authors declare no conflict of interest.

## ETHICAL APPROVAL

The study was conducted in accordance with the Declaration of Helsinki, and approved by the Ethics Committee of Beijing Shijitan Hospital (protocol code: sjtkyll‐lx‐2020(1) and date of approval: 2020‐05‐26).

## INFORMED CONSENT STATEMENT

Informed consent was obtained from all subjects involved in the study. Written informed consent has been obtained from the patient(s) to publish this paper.

## Data Availability

The data presented in this study are available on request from the corresponding author. The data are not publicly available due to restrictions, for example, privacy or ethics.
